# A research protocol for a prospective, multicenter, cohort study on interferon therapy for chronic hepatitis B combined with metabolism-associated fatty liver disease to achieve clinical cure

**DOI:** 10.3389/fpubh.2025.1546182

**Published:** 2025-03-14

**Authors:** Daqiong Zhou, Chao Zhang, Lu Zhang, Jianru Jia, Junliang Fu, Zhenhuan Cao

**Affiliations:** ^1^Beijing Youan Hospital, Capital Medical University, Beijing, China; ^2^The Fifth Medical Center of Chinese PLA General Hospital, Beijing, China; ^3^Beijing Ditan Hospital, Capital Medical University, Beijing, China; ^4^Baoding Municipal People's Hospital, Baoding, Hebei, China

**Keywords:** hepatitis B virus, chronic hepatitis B, metabolism-associated fatty liver disease, peginterferon, HBsAg clearance, clinical cure

## Abstract

**Introduction:**

The incidence of chronic hepatitis B (CHB) combined with metabolism-associated fatty liver disease (MAFLD) is increasing annually, and the presence of MAFLD may influence the clinical assessment of viral activity and transaminase levels. However, it remains unclear whether MAFLD impacts the achievement of clinical cure in CHB patients treated with polyethylene glycol interferon (Peg-IFN).

**Methods:**

A prospective cohort study was conducted to enroll patients with dominant CHB (on NA treatment, HBsAg <1,500 IU/mL, HBeAg negative, HBV DNA <10 IU/mL) and patients with dominant CHB combined with MAFLD, all of whom were treated with Peg-IFN. The study aimed to assess the efficacy and safety of Peg-IFN treatment and to elucidate the effect of MAFLD on achieving HBsAg clearance in these patients. Additionally, the study explored the T-lymphocyte characteristics of patients with CHB combined with MAFLD, analyzed the role of T-lymphocytes expressing inhibitory receptors in HBsAg clearance, and investigated the immunological mechanisms of HBsAg clearance through single-cell transcriptome sequencing technology.

**Ethics and dissemination:**

Patients will be recruited at four medical centers in Beijing and Hebei, and written informed consent will be obtained to inform participants of the purpose of the study, potential risks, and benefits. Ethical approval has been granted for the study, which will focus on 48-week HBsAg clearance, and a detailed follow-up and adverse event monitoring plan has been developed.

**Strengths and limitations of this study:**

Strengths are that this study fills the gap in treatment strategies for patients with CHB combined with MAFLD and provides important treatment guidance to clinicians; the multicenter design may increase the diversity of the sample size, reduce the bias of single-center studies, and improve the external validity of the results. Limitations are that interferon therapy is often associated with side effects, which may lead to lower patient adherence and affect long-term follow-up and outcome monitoring of the study; the heterogeneity of the MAFLD population may have different effects on the efficacy of interferon therapy.

**Clinical trial registration:**

http://www.chictr.org.cn/bin/project/edit?pid=231498, identifier ChiCTR2400084913.

## Introduction

### Background and rationale

In 2016, the World Health Organization (WHO) proposed “eliminating viral hepatitis as a major public health threat by 2030” (1). In recent years, the incidence of HBV infection has shown a significant downward trend; however, due to its large patient population, China continues to bear the highest disease burden of HBV infection globally, with 74 million affected individuals (2). This reality indicates that China remains far from meeting the WHO’s target, making the task of elimination particularly challenging.

Previous studies have concluded that HBV DNA seroclearance significantly reduces the risk of hepatocellular carcinoma (HCC) (3). However, these patients may still face the risk of reactivation. Among those with undetectable viral loads, patients with low serum HBsAg levels (<1,000 IU/mL) have the lowest risk of HCC (4). Thus, further HBsAg seroclearance is considered the best marker of HBV immune clearance. The 2022 Chinese Guidelines for the Prevention and Control of Chronic Hepatitis B emphasize that “for some patients with suitable conditions, a cure for hepatitis B should be pursued.” The guidelines further state that patients with undetectable HBV DNA, HBeAg conversion, and HBsAg <1,500 IU/mL after nucleotide analog (NA) treatment represent an advantageous population for achieving HBsAg clearance. These patients may be considered for additional treatment with polyethylene glycol interferon (Peg-IFN) in pursuit of a clinical cure (5).

In addition, non-alcoholic fatty liver disease (NAFLD) has become the most prevalent chronic liver disease in both China and globally. The most recent data report the global prevalence of NAFLD to be 30% and rising (6, 7). In 2020, the International Fatty Liver Nomenclature Panel redefined fatty liver in individuals with overweight/obesity, type 2 diabetes mellitus, or multiple metabolic disorders as metabolism-associated fatty liver disease (MAFLD) (8). In 2023, the European Annual Congress of Liver Diseases, along with the Liver Disease Associations of the United States, Europe, and Latin America, proposed that the alternative term for NAFLD should be metabolic dysfunction-associated steatohepatopathy (MASLD) (9). The prevalence of MAFLD in China was about 17.6% in 2016, with an estimated 246 million individuals affected, and is projected to rise to 29.1% by 2030, reaching 315 million cases, making MAFLD the most common chronic liver disease (10). The international expert consensus on metabolism-associated fatty liver disease notes that, with the dramatic global increase in MAFLD prevalence, it often coexists with other liver diseases, such as viral hepatitis. Large North American cohort studies have shown the prevalence of CHB combined with MAFLD to be 31.4% (11), while a single-center retrospective study in the Netherlands reported an increase from 17.2 to 24.3% in such patients (12). A recent meta-analysis of 53 studies found the prevalence of MAFLD in patients with CHB to be 32.83% (13).

Whether MAFLD affects the antiviral efficacy of hepatitis B treatments, including HBV DNA suppression, HBeAg conversion, and HBsAg clearance, remains controversial. Most current studies focus on NA treatment, with goals primarily related to HBV DNA suppression and HBeAg conversion. However, research on HBsAg clearance, an indicator of clinical cure, is limited. Notably, the impact of MAFLD on achieving clinical cure in the Peg-IFN treatment-advantaged CHB population has not yet been reported and is in urgent need of investigation. Some studies suggest that the presence of MAFLD could potentially hinder the response to interferon therapy, possibly due to alterations in immune function or liver metabolism (14). However, the findings are inconsistent, with certain studies indicating no significant impact of MAFLD on Peg-IFN efficacy (15), while others suggest that MAFLD may contribute to reduced treatment efficacy or slower rates of HBsAg clearance (16).

These conflicting results highlight a major gap in the current understanding of how MAFLD affects Peg-IFN therapy outcomes, particularly regarding HBsAg clearance, and underscore the need for further investigation. Our study aims to address this gap by evaluating the impact of MAFLD on the response to Peg-IFN treatment in chronic hepatitis B patients, specifically focusing on the achievement of HBsAg clearance as a marker of clinical cure. Additionally, the impact of hepatic steatosis on the immune status of CHB patients, such as T-lymphocyte function, has not been previously reported, and it remains unclear whether MAFLD affects clinical cure by influencing the immune status of the body.

### Objectives

In this study, a prospective clinical cohort of Peg-IFN-treated patients with advantageous CHB combined with MAFLD was established to verify the efficacy and safety of Peg-IFN in achieving HBsAg clearance. The study aims to elucidate the impact of MAFLD on HBsAg clearance in these patients and to provide practical guidance for clinicians in accurately screening suitable candidates and adjusting treatment regimens as needed. Flow cytometry was used to examine the number and function of peripheral blood T-lymphocytes expressing inhibitory receptors in both HBsAg-cleared and non-cleared patients at baseline and various time points during treatment. This was done to explore the characteristics of T-lymphocytes in CHB combined with MAFLD and to clarify the role of inhibitory receptor-expressing T-lymphocytes in HBsAg clearance. Additionally, single-cell transcriptome sequencing technology was applied to analyze changes in the gene expression profiles of immune cells in CHB patients with MAFLD treated with Peg-IFN, aiming to trace the genetic information profile changes in immune cells during HBsAg clearance and to explore the genetic-immunological mechanisms underlying HBsAg clearance.

### Trial design

**Figure 1:**
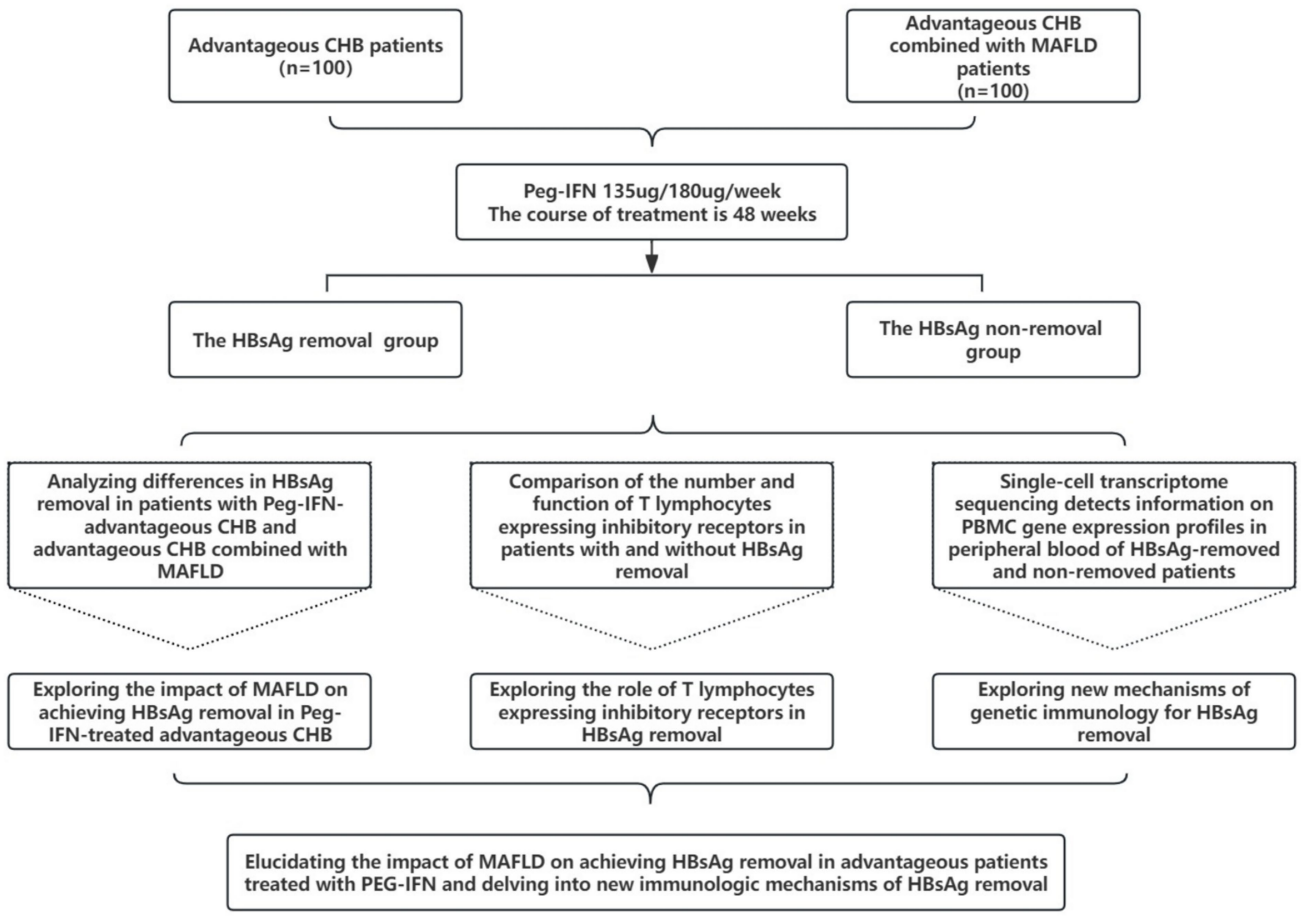
Flow chart of the research program.

This prospective study investigates the impact of MAFLD on HBsAg clearance in CHB patients treated with PEG-IFN (135ug/180ug/week for 48 weeks). Patients are divided into two groups: CHB patients (*n* = 100) and CHB with MAFLD patients (*n* = 100). After treatment, participants are categorized into HBsAg clearance responders and non-responders. For HBsAg responders and HBsAg non-responders, analyzes differences in HBsAg clearance rates between CHB and CHB with MAFLD patients. Explores the impact of MAFLD on HBsAg clearance mechanisms using IHC and other methods. Compares the immunosuppressive microenvironment between responders and non-responders, focusing on T-cell characteristics. Identifies immune mechanisms contributing to HBsAg clearance. The flow chart of this study is shown in Figure 1.

## Methods: subjects, interventions, and outcomes

### Study setting

This prospective, multicenter, open cohort study will include HBV-infected patients from four large tertiary hospitals in North China. A total of 200 participants are planned, comprising 100 patients with CHB and 100 patients with CHB combined with MAFLD. We used the SPIRIT checklist when writing our report (17). The Institutional Review Board of Beijing You’an Hospital affiliated with Capital Medical University approved the trial protocol (version 9.1, reference number LL-2024-051-K, dated May 10, 2024).

### Eligibility criteria

**Figure 2:**
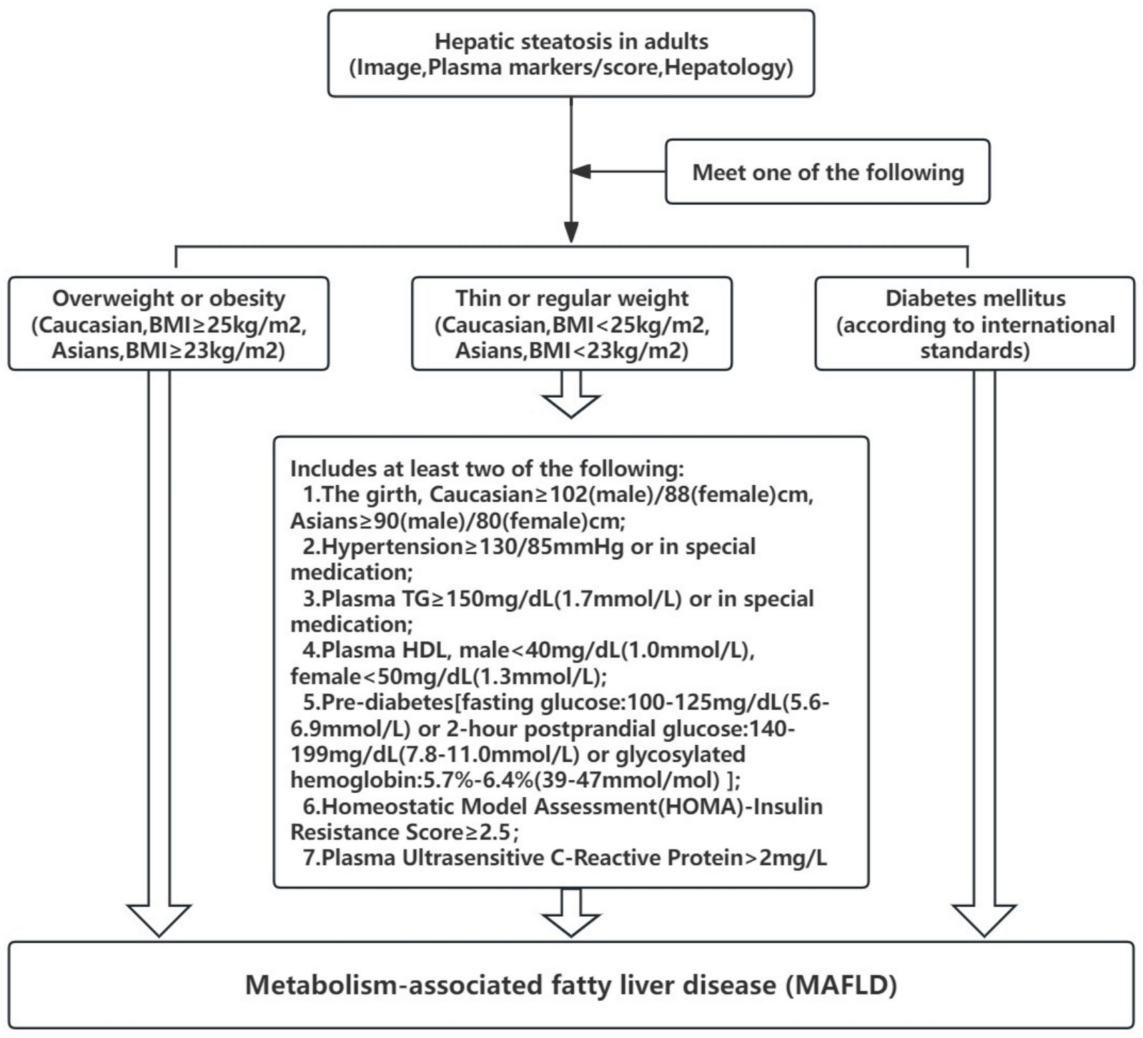
MAFLD diagnostic process and criteria.

Inclusion Criteria:

1. Chronic Hepatitis B (CHB): Meets the 2022 Guidelines for the Management of Chronic Hepatitis B: (1) HBsAg positive for >6 months; (2) On NA treatment; (3) HBsAg <1,500 IU/mL; (4) HBeAg negative; (5) HBV DNA <10 IU/mL.
2. Metabolism-Associated Fatty Liver Disease (MAFLD):

(1) Diagnosis of hepatic steatosis confirmed by abdominal ultrasound, Fibroscan, or liver steatosis measurement via MRI. Steatosis is categorized as mild, moderate, or severe based on the thresholds of the above tests. (2) Additionally, the patient must meet one of the following three conditions: ①. Overweight or obesity (BMI ≥ 23 kg/m^2^); ②. Diabetes mellitus; ③. Lean or normal-weight patients meeting at least 2 metabolic abnormalities (e.g., Figure 2).

Exclusion Criteria: (1) Patients with combined HCV infection or autoimmune liver disease; (2) History of heavy alcohol consumption (alcohol intake >210 g/day for men and > 140 g/day for women); (3) Drug-induced secondary hepatic steatosis (e.g., tamoxifen, hormones); (4) Pregnant or lactating women, and those planning to conceive shortly; (5) Patients with a history of or current cirrhosis or hepatocellular carcinoma; (6) Comorbidities with other serious diseases that may affect nutritional status, such as malignant tumors, severe cardiac, pulmonary, renal, or other organic or psychiatric diseases; (7) Neutrophil counts <2 × 10^9^/L and/or platelet counts <80 × 10^9^/L, and total bilirubin >34 μmol/L before treatment.

### Intervention description

1 Peg-IFN dosing:
(1) For patients with body weight ≥ 70 kg: 180 μg once a week, subcutaneous injection.
(2) For patients with body weight < 70 kg: 135 μg once a week, subcutaneous injection.
2 Duration:
(1) The treatment duration is 48 weeks.
(2) If the patient achieves HBsAg serologic conversion (HBsAb >300 IU/L) and maintains this response for 24 weeks, the treatment can be discontinued for observation, even if the full 48 weeks have not been completed.

### Criteria for discontinuing or modifying allocated interventions

Discontinuation Due to Special Requirements: If the patient requires discontinuation of Peg-IFN due to special circumstances (e.g., trauma, surgery, personal preference), the treatment can be resumed once the conditions for using Peg-IFN are met again. Serious Adverse Events (SAEs): If SAEs occur, the necessity to discontinue Peg-IFN will be assessed, and treatment will be adjusted accordingly.

### Strategies to improve adherence to the intervention

In this study, we provided around-the-clock (24-h) health consultation services to ensure that patients could access professional support and guidance from healthcare providers at any time. Our team offered continuous attention and necessary assistance throughout the study, addressing a range of needs including study-related treatment guidance, management of adverse drug reactions, monitoring of virological breakthroughs, and general health inquiries from patients.

### Relevant concomitant intervention permitted or prohibited during the trial

Adverse effects of PEG-IFNα-2b include transient, early-onset flu-like symptoms such as fatigue, fever, muscle aches, headache, chills, and joint pain, which typically resolve within a few days to a few weeks. Some patients may experience reduced appetite, nausea, abdominal pain, and diarrhea. Additional symptoms include dizziness, hair loss, weight loss, visual disturbances, dry and itchy skin, injection site discomfort, and rashes. Furthermore, patients may encounter cognitive and psychological issues, such as difficulty concentrating, memory impairment, anxiety, irritability, and sleep disorders. Temporary changes in laboratory parameters, including decreases in white blood cell, neutrophil, and platelet counts, as well as alterations in liver biochemical function, have also been observed. PEG-IFNα-2b can cause thyroid dysfunction, exacerbate diabetes, or induce certain immune system disorders. To address these adverse effects, a dedicated team conducts regular follow-ups and provides timely interventions. Management strategies for adverse effects may involve discontinuing therapy, dose modifications, or targeted supportive treatments for specific side effects.

### Outcome

Primary outcome indicators: 48-week HBsAg clearance rate. Secondary outcome indicators: 48-week HBsAg quantitative decline, HBV DNA undetectable rate, HBsAg serologic conversion rate, Peg-IFN adverse reactions.

### Participant timeline

**Figure 3:**
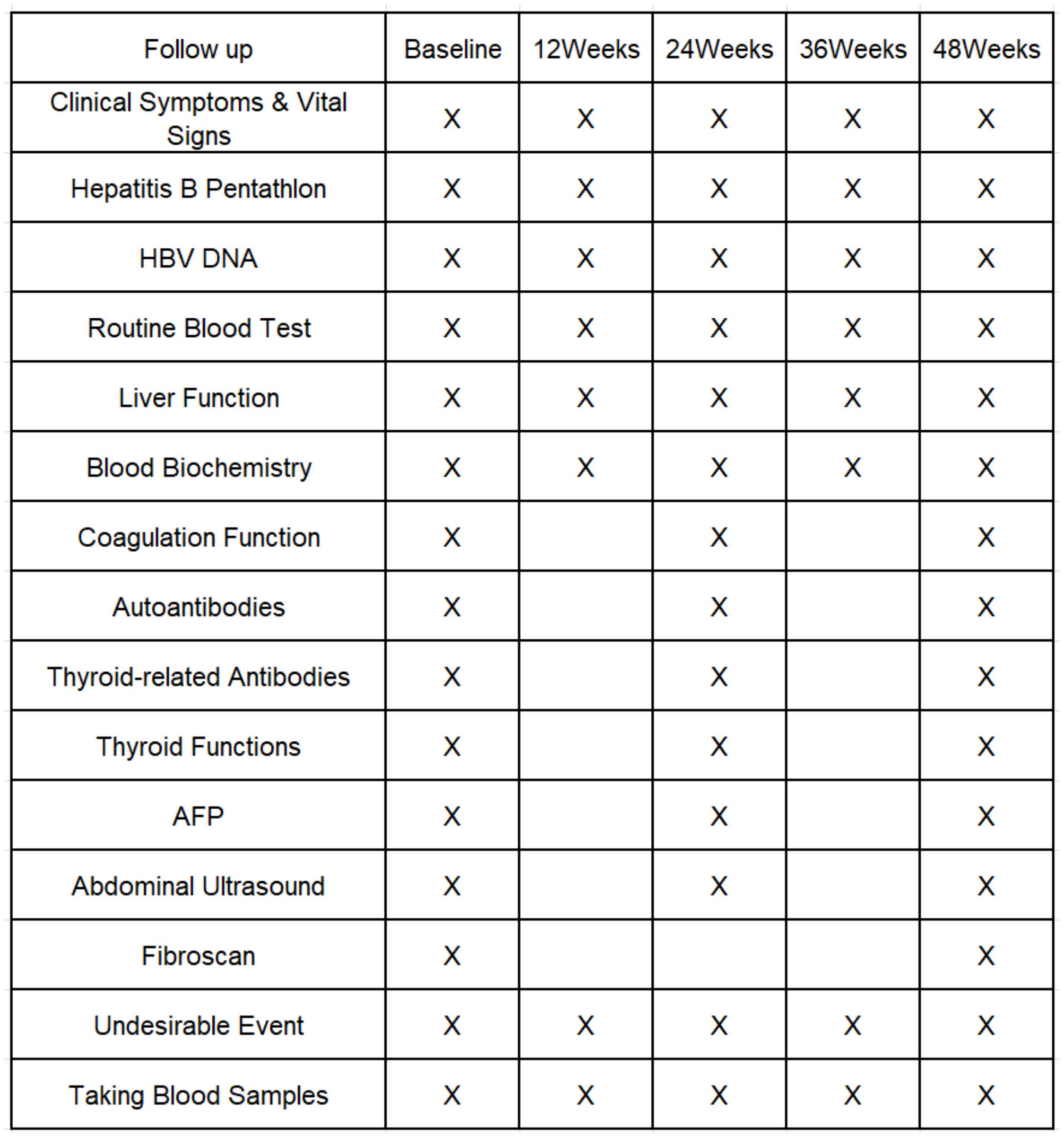
Schedule of enrollment and follow-up.

A Case Report Form (CRF) form will be established and completed for each enrolled patient, with follow-up visits scheduled every 12 weeks (±2 weeks) at baseline and throughout the treatment period. Clinical symptoms and signs will be observed during each follow-up visit, and peripheral blood PBMC and plasma specimens will be collected and stored at −80°C for future research. The clinical test items at the follow-up visit are shown in Figure 3.

### Sample size

Since there are no existing literature reports on Peg-IFN treatment for combined MAFLD in the CHB-advantaged population, the sample size for this study is based on the results of our team’s previous research. In that study, Peg-IFN treatment of CHB and CHB combined with MAFLD patients for 48 weeks resulted in HBsAg clearance rates of 35 and 60%, respectively. Given the lack of established data in this specific population, we chose to use this estimate as it is reflective of our previous work, which involved similar patient profiles and treatment conditions. Setting the test level at *α* = 0.05 and the power of the study (1-*β*) at 0.90, the required sample size was calculated using the test module for two independent sample rates in PASS 11.0. The calculation indicated that 79 patients should be enrolled in each group. Considering a 15% dropout rate, 92 participants are needed in each group, thus expanding the sample size to 100 cases per group.

### Recruitment

Participants will be recruited from four medical centers in Beijing and Hebei, China: Beijing You’an Hospital of Capital Medical University, Fifth Medical Center of General Hospital of the People’s Liberation Army, Beijing Ditan Hospital of Capital Medical University, and Baoding People’s Hospital. Each hospital will have a study coordinator to facilitate and coordinate the trial. Patients will be informed of the study’s purpose, procedures, and potential risks and benefits. Written informed consent will be obtained from all participants. They will also be allowed to withdraw from the trial at any time without any consequences.

Patients will be invited to participate in this study shortly after diagnosis. At the screening follow-up, a clinically experienced researcher (physician) will confirm eligibility for enrollment by reviewing symptoms, laboratory results, and imaging such as ultrasound or CT, ensuring that each inclusion and exclusion criterion is met. Recruitment will occur over 24 months, from January 2024 to December 2025.

No patients or members of the public other than professional clinicians and researchers were involved in the design, conduct, reporting, or dissemination plans for this study.

## Methods: data collection, management, and analysis

### Plans for assessment and collection of outcomes

Data will be collected using specially designed paper-based CRFs. Once completed, these forms will be entered into a database by designated personnel after prior data inspection. The Schedule of enrollment and follow-up (Figure 3) provides a detailed illustration of both the schedule and the specific nature of data collection required throughout the study period.

### Plans to promote participant retention and complete follow-up

Upon study completion, if a patient achieves HBsAg seroclearance, this outcome is considered the optimal result approaching “clinical cure,” indicating significantly improved long-term prognosis. For such cases, our research team will develop a follow-up monitoring and health management plan to maintain the therapeutic effect. Conversely, if HBsAg seroclearance is not achieved, clinicians will tailor the treatment strategy based on the patient’s specific health condition, aiming to optimize therapeutic outcomes and continue to control disease progression. Regardless of treatment outcomes, we are committed to providing patients with the most suitable personalized follow-up care to ensure they receive optimal health support.

### Data management

The number of sample cases in this subject is large, and the nodes of retained specimens and test items are more, in order to ensure the uniformity and accuracy of the test, we take the following measures: in the process of specimen retention and separation, there are people responsible for the registration, sampling, separation of cells and serum, and freezing; specimens retained at different time nodes are uniformly detected by the specialized inspectors to ensure the reliability and accuracy of the test results. Trial data were collected and stored in accordance with GCP guidelines [China National Medical Products Administration, National Health Committee. Notice on the publication of Good Clinical Practice (no. 57 of 2020). April 26, 2020 (in Chinese). https://www.nmpa.gov.cn/directory/web/nmpa/xxgk/ggtg/ypggtg/ypqtggtg/20200426162401243.html]. Final approval of the research database will only be granted after successful implementation of these protocols has been confirmed.

### Statistical methods for primary and secondary outcomes

#### Primary outcomes

The Kaplan–Meier method was used to estimate the cumulative incidence of HBsAg seroclearance, and the log-rank test was used to compare the cumulative probabilities of different subgroups.

#### Secondary outcomes

A linear mixed-effects model was employed to analyze the trends in HBsAg levels over time. Random effects were included to account for inter-individual variability among patients. Fixed effects in the model included time (measured in months), group classification (e.g., responder vs. non-responder, MASLD group vs. non-MASLD group, male vs. female), and the interaction between time and group classification. Additionally, marginal effects of changes in HBsAg levels over time for different groups were estimated using generalized estimating equations (GEE). This approach provided a comprehensive assessment of the temporal variation in HBsAg levels across various patient subgroups.

### Methods for any additional analyses

SPSS 27.0 statistical software was used for data analysis. Categorical variables were expressed as counts and percentages, while continuous variables were expressed as mean and standard deviation. Clinical data were statistically analyzed using the Kruskal-Wallis test. Variance homogeneity across groups was assessed for each test index. If the variance was homogeneous, the LSD method of one-way ANOVA was applied; if the variance was not homogeneous, the Kruskal-Wallis test was used for comparisons across multiple groups. For comparisons between the two groups, the Mann–Whitney *U* test was employed. Immunological data, including HBsAg levels, HBV DNA titers, and immune marker levels (e.g., T-cell responses, cytokine profiles), will be analyzed using appropriate statistical methods based on the data distribution. Descriptive statistics (mean, SD, median, and IQR) will be used to summarize the data. For longitudinal analysis of immune markers (such as HBsAg clearance and T-cell responses) over the course of treatment, mixed-effects models will be employed to account for repeated measures and correlations within subjects. If immunological data are not normally distributed, non-parametric tests such as the Mann-Whitney U test (for comparisons between two independent groups) or the Kruskal-Wallis test (for comparisons across more than two groups) will be used. Cox proportional hazards regression will be utilized to assess the relationship between immunological markers (e.g., HBsAg clearance, T-cell response) and clinical outcomes, such as sustained HBsAg seroclearance or progression to clinical cure. In addition, we will conduct correlation analyses (Spearman’s rank correlation) to explore potential associations between immune parameters (e.g., cytokine levels, T-cell counts) and clinical variables (e.g., liver function, ALT/AST levels). A *p*-value of <0.05 was considered statistically significant.

### Methods used to analyze population and missing data

The missing values of all variables were adjusted by applying Multiple Imputation (MI). If more than 15 per cent of data points are missing, sensitivity analyses of the main results are conducted using multiple imputation techniques. If the missing data is determined to be not missing at random (NMAR), we will explore sensitivity analyses to assess the impact of different assumptions about the missing data mechanism. We will also provide a drop-out analysis to evaluate any potential bias introduced by participant attrition.

## Methods: monitoring

### Data monitoring committee (DMC)

This project adopts a three-tier management system involving the principal investigator, the hospital’s Research Department, and the hospital leadership. The principal investigator is responsible for the implementation and management of the project, coordinating periodic inspections and supervision conducted by the Beijing Municipal Health Commission and the hospital. Regular progress reports will be submitted to the higher-level supervisory departments and the hospital’s Research Department. Upon completion of the project, the principal investigator will assist relevant supervisory bodies in organizing the acceptance and conclusion of the project in accordance with the implementation plan.

### Interim analyses

The superior CHB cohort and the CHB combined MEFLD cohort continued to be maintained between 2025–01 and 2025–12. Regular follow-up observations were made every 12 weeks to record HBsAg and HBsAb, HBeAg and HBV DNA data and adverse events. For some of those who completed 48 weeks of antiviral therapy, the difference in efficacy between the two groups was initially analyzed.

### Adverse event reporting

All serious adverse events (SAEs) or unexpected incidents that may impact the safety or rights of study participants must be reported within 24 h of detection by the research personnel at the study institution. A comprehensive incident report must be submitted to the principal investigator and the relevant regulatory authorities. Following the report, the coordinating center will oversee the initiation of an investigation by the Ethics Committee supervising drug-related affairs to determine the cause of the adverse event. All SAEs must be thoroughly documented in the participant’s medical records or trial files. This documentation must be signed and dated by the investigator responsible for the participant to facilitate future audits and monitoring activities. Furthermore, all reported adverse events must be communicated to the Ethics Committee regularly, at intervals of 6 months, in accordance with the specific requirements of the Ethics Committee.

### Frequency and procedures for auditing trial conduct

In this study, each participating research institution will assign two independent researchers to conduct an initial review and verification of the standardized reporting format (CRF) to ensure accuracy and completeness. Following the preliminary review, a data manager will perform centralized verification to identify and correct any discrepancies or errors, with assistance from clinical monitors before the data is finalized. Additionally, a designated auditor, independent of both the investigators and the principal investigator, will conduct regular audits of the standardized reporting format at each research institution. Throughout the trial, the CRFs will undergo routine review every 6 months to ensure data integrity and compliance.

## Ethics and dissemination

### Protocol amendments and approvals

Any changes to the study protocol that may affect the study’s progress, the potential benefits to participants, or the safety of participants—such as modifications to the study objectives, design, patient demographics, sample size, procedures, or significant management details—must be formally revised. These amendments must receive approval from the Trial Steering Committee and the Ethics Committee before implementation.

Modifications proposed due to trial execution must be submitted by the principal investigator and discussed with the Multicenter Coordination Committee. These proposed amendments should be documented and signed by the principal investigator and relevant centers. The modifications can only be implemented after receiving formal approval from the Ethics Committee.

### Informed consent

In this study, we will thoroughly communicate with the patients to emphasize the importance of antiviral treatment and the significant impact of HBsAg clearance on improving patient prognosis. Informed consent will be obtained from each patient.

### Additional consent provisions

Patients will be required to visit the hospital for assessments at baseline, Week 12, Week 24, Week 36, and Week 48 after enrollment. Routine examinations will include blood routine tests, liver function biochemistry, thyroid function, HBsAg quantification, and HBV DNA levels. Additionally, 20 mL of blood will be collected for immunological and host genetic susceptibility marker analysis.

### Patient medical record confidentiality

The patient’s medical records will be kept securely and in full at the hospital where the patient receives treatment. The physician will document the results of laboratory tests and examinations in the patient’s medical records. Any public reports related to the results of this study will not disclose any personal information about the patients. We will make every effort, within the limits of the law, to protect the privacy of the patient’s personal medical information.

### Financial management of research funds

In strict accordance with the financial management regulations for research projects established by the Beijing Municipal Health Commission’s First Fund, the use of research funds will be applied for by the project leader, signed, recorded by the hospital’s research office, and approved by hospital leadership before being specifically allocated or reimbursed by the finance department. The project leader will regularly check and supervise the research progress and the usage of funds. Based on the project’s implementation, the project leader will propose adjustments to the funding and submit them for review and filing with the hospital’s finance department and research office.

### Management of adverse events and health changes during the study

If, during the study, a patient experiences any discomfort, new changes in their condition, or any unexpected events, whether related to the study or not, they must promptly notify the relevant research center physician. The physician will assess the situation and provide appropriate medical treatment. The treatment measures may include discontinuation of medication, dose reduction, or other supportive therapies. Adverse reactions during interferon therapy are considered normal; most symptoms will resolve after appropriate management. The research team will closely monitor these reactions and provide timely and appropriate interventions.

### Plan for dissemination of research findings

By the applicant’s previous research foundation and domestic and international literature, this prospective clinical study is expected to obtain a high HBsAg clearance rate, enable more patients with hepatitis B to obtain the clinical cure, greatly improve the prognosis of the patients, which can significantly reduce the transmission of HBV, reduce the incidence of cirrhosis and hepatocellular carcinoma, reduce the long-term expenditures, save more labor force, and create higher socio-economic benefits. It will provide evidence-based medical evidence for the updating of China’s chronic hepatitis B prevention and treatment guidelines. The specific research area of this topic is attributed to viral hepatitis. One of the promotion methods is to promote the experience gained in clinical and basic research of PEG-IFN treatment of advantageous CHB to colleagues in the field of hepatology through academic lectures or international and domestic academic conference exchanges. Secondly, we will publish the important experience gained from our research in the form of papers for reference by our colleagues; thirdly, we will organize a workshop on hepatitis B antiviral treatment to promote our experience.

## Discussion

The prevalence of MAFLD is steadily increasing both in the general population and among patients with CHB worldwide. Although fatty liver is recognized as a risk factor for adverse liver outcomes, including cirrhosis and hepatocellular carcinoma, its interactions with HBV and the resulting clinical effects remain complex and not fully understood.

Some studies have identified fatty liver as an unfavorable factor affecting antiviral efficacy. In a study of 213 Chinese patients with CHB treated with entecavir (ETV), the rate of HBV DNA undetectability at 24 weeks was significantly lower in patients with comorbid steatosis compared to those without hepatic steatosis (47.7% vs. 58.8%, *p* = 0.01) (18). Additionally, in a study of 89 HBeAg-positive CHB patients treated with Peg-IFN for 48 weeks, those with comorbid steatosis had significantly lower HBV DNA undetectability rates 48 weeks after discontinuation compared to those without steatosis. Multifactorial analysis showed that steatosis was an unfavorable factor for sustained viral response (OR = 0.012, *p* = 0.02) (19). The EFFORT study suggests that new-onset and persistent MAFLD in patients with HBeAg-positive CHB treated with NA may counteract the benefits of antiviral therapy, reduce the rate of ALT normalization, and negatively influence fibrosis improvement (20).

In contrast, some studies have shown that patients with hepatic steatosis are more likely to achieve HBsAg clearance. The metabolic components and immune alterations associated with MAFLD progression may directly inhibit HBV replication or indirectly induce antiviral immune responses. In patients with chronic HBV infection, comorbid MAFLD has been found to inhibit HBV replication and is associated with spontaneous HBsAg clearance (21). An international multicenter randomized controlled study of 4,769 patients with treatment-naïve CHB, treated with either entecavir (ETV) or tenofovir (TDF), with a median follow-up time of approximately 5.16 years, found that 58 patients achieved HBsAg clearance, and hepatic steatosis was identified as a favorable factor for HBsAg clearance (HR = 1.84) (22). Additionally, a previous study by our group showed that the 96-week HBsAg clearance rate was higher in inactive HBsAg carriers with moderate steatosis compared to those without steatosis (23).

In contrast, some studies have shown that steatosis is not associated with antiviral efficacy. In a retrospective study of 555 CHB patients treated with lamivudine (LAM), adefovir (ADV), entecavir (ETV), or tenofovir (TDF), multivariate analysis revealed no significant difference in HBV DNA suppression between patients with or without comorbid NAFLD (24). Another retrospective study from Taiwan involving 196 patients with HBeAg-positive CHB treated with NA monotherapy (LAM, ADV, LdT, ETV, or TDF) found that steatosis had no significant effect on HBeAg clearance (54.9% vs. 57.4%) (25).

There are differing conclusions and opinions on whether MAFLD affects antiviral efficacy. Most current studies have primarily focused on NA treatment, with therapeutic targets related to HBV DNA suppression and HBeAg conversion. However, studies on HBsAg clearance, the indicator of clinical cure, are rare. Notably, it remains unreported whether MAFLD affects the clinical cure in patients with predominantly CHB undergoing Peg-IFN treatment. This study aims to elucidate the impact of MAFLD on the clinical cure of dominant CHB patients, with the hope of guiding clinicians to more accurately screen suitable patients and promptly adjust treatment regimens.

T lymphocytes play a crucial role in the progression of HBV disease, including viral clearance, the development of hepatitis, cirrhosis, and hepatocellular carcinoma, as well as in response to antiviral therapy. CD8+ T cells are particularly important, as they can induce apoptosis in virus-infected hepatocytes. Additionally, they inhibit HBV gene expression and replication in hepatocytes through a non-cytolytic mechanism by secreting IFN-*γ*. This dual function of CD8+ T cells—mediating apoptosis and inhibiting viral replication—highlights their essential role in controlling HBV infection. When HBV infection becomes chronic, T cells gradually become dysfunctional and lose their immune efficacy as the infection progresses and antigen exposure continues. This dysfunction is characterized by a decrease in the secretion of cytotoxic factors, such as IL-2, IFN-γ, and TNF (26, 27), and an increase in the secretion of immunosuppressive factors like IL-10 and transforming growth factor-beta (TGF-*β*). Additionally, there is increased expression of inhibitory receptors such as programmed death protein 1 (PD-1), cytotoxic T-lymphocyte-associated antigen-4 (CTLA-4), and T-cell immunoglobulin and mucin structural domain-3 (Tim-3) (28, 29). Inhibitory receptors are likely the primary reason for the loss of T-lymphocyte function, leading to the body’s inability to effectively clear HBV (30). A therapeutic strategy that rescues functionally exhausted T cells by blocking inhibitory receptor signaling pathways, thereby enhancing the immune response, could integrate immunotherapy with antiviral therapy. This approach holds promise as a reliable option for patients with chronic HBV infection to achieve a functional cure.

Recent studies have reported that hepatic CD8+ T cells in MAFLD mice may play a role in alleviating hepatic inflammation and fibrosis (31). Additionally, the number of peripheral blood Th1 cells in patients with MAFLD is significantly higher than in healthy individuals, leading to the hypothesis that Th1 cells may have a pro-inflammatory role (32). However, the state of T-lymphocyte immunocompetence in patients with CHB combined with MAFLD has not yet been reported.

Based on a cohort of CHB or CHB-combined MAFLD patients receiving Peg-IFN antiviral therapy, along with a comprehensive clinical database and specimen bank, this study aims to investigate the number and function of T lymphocytes expressing inhibitory receptors in the peripheral blood of HBsAg-cleared and non-cleared patients at various time points, both at baseline and during treatment, using flow cytometry. The goal is to characterize the T-lymphocytes in patients with CHB combined with MAFLD, clarify the role of T lymphocytes expressing inhibitory receptors in HBsAg clearance, explore potential therapeutic targets, and provide insights for the development of new drugs aimed at achieving a clinical cure for hepatitis B.

Sequencing of immune cells, such as T lymphocytes and DC cells, from liver cancer patients has revealed the characteristics of immune cells within the liver cancer environment, aiding in the development of more effective immunotherapeutic targets (33). However, the application of single-cell sequencing technology in populations with CHB combined with MAFLD has not yet been explored, particularly in investigating the mechanisms involved in HBsAg clearance. In this study, we aim to use transcriptome single-cell sequencing to explore the immunomolecular mechanisms of HBsAg clearance in CHB patients with MAFLD who are receiving Peg-IFN treatment. This approach will provide a multi-stage, dynamic, and high-resolution immune landscape of the HBsAg clearance process, clarify the gene regulation of immune cells during HBsAg clearance, uncover the immune cytogenetic basis of this clearance, and identify the molecular immunogenetic markers that determine successful HBsAg clearance. In addition, although this study has adopted a prospective and multicenter study protocol to reduce sampling and follow-up bias, there are still some limitations: (1) Given that the study is multicenter and cohort-based, there may be variability in treatment outcomes due to differences in patient populations, hospital settings, and clinician practices. While we aim to include a representative sample, the findings may not be generalizable to all HBV patients, particularly those outside of the study centers or those with more advanced liver disease. (2) The study relies on patient adherence to treatment protocols, including Peg-IFN therapy, and the potential for non-compliance or early dropout could impact the results, particularly the achievement of HBsAg clearance. (3) While we will attempt to control for common confounding factors such as age, gender, and baseline liver function, there may be other unmeasured factors (e.g., genetic predispositions, lifestyle habits, or co-infections) that could influence the treatment response and the success of Peg-IFN therapy.

## Experimental state

The study has received all approvals from the Ethics Committee to start in all participating hospitals. The study was registered on May 28, 2024 (ChiCTR2400084913). Patient enrollment started on June 1, 2024.

Timeline and Milestones:

By December 2024:

(1) Develop and finalize various SOPs.
(2) Enroll eligible CHB patients.
(3) Peg-IFN combined with NA therapy should be administered to both the dominant CHB population and the CHB combined with MAFLD patients.
(4) Conduct regular follow-ups to monitor patient progress.

January 2025 to December 2025:

Complete the enrollment of all patients.

January 2026 to December 2026:

(1) Analyze and compare the number and function of inhibitory receptor T-lymphocytes between the two groups.
(2) Assess differences in HBsAg clearance rates.
(3) Compare the number and function of inhibitory receptor T-lymphocytes between HBsAg-cleared and non-cleared patients.
(4) Use single-cell sequencing technology to analyze the mechanisms underlying HBsAg clearance.
